# *Pyrococcus furiosus* flagella: biochemical and transcriptional analyses identify the newly detected *flaB0* gene to encode the major flagellin

**DOI:** 10.3389/fmicb.2014.00695

**Published:** 2014-12-11

**Authors:** Daniela J. Näther-Schindler, Simone Schopf, Annett Bellack, Reinhard Rachel, Reinhard Wirth

**Affiliations:** ^1^Institute of Microbiology and Archaea Center, University of RegensburgRegensburg, Germany; ^2^Plant Development, Department of Biology I, Biocenter of the Ludwig Maximilian University of MunichPlanegg-Martinsried, Germany; ^3^Department of Biology - Section Environmental Microbiology, Technical University FreibergFreiberg, Germany

**Keywords:** archaeal flagella, *Pyrococcus furiosus*, Fla proteins, major flagellin, FlaB0, transcriptional analyses

## Abstract

We have described previously that the flagella of the Euryarchaeon *Pyrococcus furiosus* are multifunctional cell appendages used for swimming, adhesion to surfaces and formation of cell-cell connections. Here, we characterize these organelles with respect to their biochemistry and transcription. Flagella were purified by shearing from cells followed by CsCl-gradient centrifugation and were found to consist mainly of a ca. 30 kDa glycoprotein. Polymerization studies of denatured flagella resulted in an ATP-independent formation of flagella-like filaments. The N-terminal sequence of the main flagellin was determined by Edman degradation, but none of the genes in the complete genome code for a protein with that N-terminus. Therefore, we resequenced the respective region of the genome, thereby discovering that the published genome sequence is not correct. A total of 771 bp are missing in the data base, resulting in the correction of the previously unusual N-terminal sequence of flagellin FlaB1 and in the identification of a third flagellin. To keep in line with the earlier nomenclature we call this *flaB0*. Very interestingly, the previously not identified *flaB0* codes for the major flagellin. Transcriptional analyses of the revised flagellar operon identified various different cotranscripts encoding only a single protein in case of FlaB0 and FlaJ or up to five proteins (FlaB0-FlaD). Analysing the RNA of cells from different growth phases, we found that the length and number of detected cotranscript increased over time suggesting that the flagellar operon is transcribed mostly in late exponential and stationary growth phase.

## Introduction

Archaea have been shown to possess various distinct types of cell surface appendages (reviewed e.g., by Ng et al., 2008 or Jarrell et al., 2013) of which flagella are the best characterized ones. Superficially, these structures seem to be very similar to bacterial flagella; however, analyses of the ultrastructure, the involved proteins and the biosynthesis machinery identified fundamental differences (see e.g., Thomas et al., 2001 or Ghosh and Albers, 2011 for reviews on archaeal flagella). Based on these findings a renaming of archaeal flagella into archaella has been suggested (Jarrell and Albers, 2012) but has been questioned because of serious flaws as consequences of such a nomenclature (Wirth, 2012). With respect to the ongoing discussion about a name reflecting the function and uniqueness of these cell surface structures, we decided to retain the term flagella.

Archaeal flagella are built in their part emanating from the cell from mostly more than one protein, the so-called flagellins. *In silico* analyses of many different archaeal genomes found that the genes encoding flagellins (*flaA* and/or *flaB*) are arranged in an operon together with additional proteins assumed to be motor and anchoring components. In Euryarchaeota, the operon comprises the *fla*-associated genes *flaC* to *flaJ*, whereas in Crenarchaeota, *flaCDE* are missing and *flaX* is present (which is absent from Euryarchaeota). Interestingly, neither these genes nor the corresponding proteins show any similarities to their bacterial counterparts (Jarrell et al., 2013). Hence, our current knowledge of the assembly of archaeal flagella is based on genetic analyses. Deletion studies in *Halobacterium salinarum, Methanococcus maripaludis*, and *Sulfolobus acidocaldarius* have shown that all of the *fla*-associated genes are necessary for proper assembly and function of flagella (Patenge et al., 2001; Chaban et al., 2007; Lassak et al., 2012). Flagellins are synthesized as preproteins; their signal peptide is removed by FlaK and an N-linked glycan is attached by the oligosaccharyltransferase AglB. These posttranslationally modified subunits are supposed to be incorporated at the base of the growing non-tubular structure involving the activity of the ATPase FlaI and the conserved membrane protein FlaJ (Jarrell et al., 2013).

In addition to the mentioned mesophilic and thermophilic species, the Euryarchaeon *Pyrococcus furiosus* is a model organism for hyperthermophilic Archaea. Despite the availability of a genetic system (Waege et al., 2010; Lipscomb et al., 2011) and numerous –omics-based approaches (for a summary see Bridger et al., 2012), data on its flagella are restricted to a publication of our group (Näther et al., 2006). We have shown that *P. furiosus* uses its flagella not only for swimming, but is able to adhere with these cell surface organelles to specific surfaces including cells of its own species, thereby forming biofilms. In addition, also the formation of cell-cell connections via cable-like aggregated flagella was observed (Näther et al., 2006). In further studies we have demonstrated that also the flagella of the fastest organisms on earth (Herzog and Wirth, 2012), namely the Euryarchaeon *Methanocaldococcus villosus*, can be used for adhesion to various surfaces; again, also formation of cell-cell connections by flagella was described (Bellack et al., 2011). Beside the functional studies, we have proven that the flagella of *P. furiosus* consist of mainly one glycoprotein (Näther et al., 2006), but the N-terminal sequence we identified did not match perfectly to any protein annotated in the published genome sequence (Robb et al., 2001). Therefore, we resequenced the flagellar operon in this study and discovered that a 771 bp segment was missing previously in the genome sequence. On this segment, we identified an in-frame start codon for the *flaB1* gene and a new gene, *flaB0*, which encodes the major flagellin. In addition we performed *in vitro* polymerization studies of flagellin monomers and analyzed transcription of the revised flagellar operon of *P. furiosus*.

## Materials and methods

### Growth of *P. furiosus*, flagella preparation, and repolymerization of denaturated flagellins

Growth of cells and preparation of flagella therefrom by shearing followed by CsCl-gradient centrifugation was as described (Näther et al., 2006). For repolymerization studies, flagella were isolated as follows: cells were lysed by osmotic shock; membranes were then harvested by differential centrifugation and solubilized overnight at room temperature by addition of 0.5% n-dodecyl β-D-maltopyranoside (DDM). After purification by CsCl-gradient centrifugation (Näther et al., 2006), flagella were denatured by addition of SDS to a final concentration of 1% and heating at 100°C for 30 min. The samples were dialyzed four times for 1 h each against 5 mM HEPES buffer (pH = 7.0), followed by an overnight dialysis. These samples were incubated in tightly closed vials at various temperatures (8°C, 37°C, 60°C, and 90°C) with/without addition of 1 mM ATP. To avoid evaporation samples incubated at 60°C and 90°C were overlaid with chill-out liquid wax (*Biorad Laboratories GmbH; Munich, Germany*). Aliquots were analyzed by SDS-PAGE after 1, 2, and 6 days, without heating prior to loading.

N-terminal sequencing by Edman degradation was performed by the protein analytic facility of the Biochemistry Department of the University of Regensburg.

### DNA isolation and sequencing

For DNA isolation cells were collected from 40 ml cultures by centrifugation and DNA was isolated according to Bellack et al. (2011). Alternatively, cells were resuspended in 0.8 ml TNE buffer (100 mM Tris/Cl; 50 mM NaCl; 50 mM EDTA; pH = 8.0). Lysis of cells was by addition of 0.1 ml of 10% SDS plus 0.1 ml of 10% N-lauroylsarcosine and cautious mixing. After addition of 10 μl RNase (10 mg/ml) and incubation for 15 min at room-temperature 50 μl proteinase K (20 mg/ml) was added and the sample heated for 1 h to 55°C. After repeated phenol extractions DNA was precipitated from the water phase with 800 μl 2-propanol, the pellet was washed with 70% ice-cold ethanol and dissolved in water.

For resequencing of the genomic region around the *flaB2* gene, primer walking analyses were performed using primers 353420_f_ (5′-ATGGAAAAACTAGAGAAGACCGTTG-3′), 352920_f_ (5′-TGGCTCAGCTTCACCAGC-3′), 352542_f_ (5′-AATATTAGATGAGGGATTCGAAGTTAA-3′), 352509_f_ (5′-GGATTATGGAAAGGCAATTCTTCTC-3′), 353159_r_ (5′-TATTGCCATCTTAACTATGGTCCC-3′), and 351761_r_ (5′-ATCACATTATACTCAAATGTTGGGG-3′). Primer numbers refer to the binding position in the original genome sequence (Robb et al., 2001).

PCR reactions using primers 353483_f_ (5′-GGATTATGGAAAGGCAATTCTTCTC-3′) and 351761_r_ were used to analyze genomic DNA from various *P. furiosus* strains for the presence of the *flaB0* gene.

### Generation of antibodies

To raise specific antibodies against each flagellin, the respective central region (**Figure 2**, gray sequences) was amplified via PCR using primers FlaB0-MT_f_ (5′-GGATCCGAGAAAACAGCATATCACAAAGGA-3′), FlaB0-MT_r_ (5′-AAGCTTACCGAAAACTCCATTTCCCT-3′), FlaB1-MT_f_ (5′-GGATCCAGTGGAGAACTGTACACTGGAAAGA-3′), FlaB1-MT_r_ (5′-AAGCTTGCTCTTATAATTAAAGACATCATCCGT-3′), FlaB2-MT_f_ (5′-GCAGCCATATGAGGTATTACGATCCA-5′), and FlaB2-MT_r_ (5′-GAAGGGGATCCTCAGTAGAGGTTCCA-5′). Fragments were cloned into the low-copy number plasmid pQE30 (*QIAGEN; Hilden, Germany*) to avoid instable clones. The plasmid was transformed into the *E. coli* expression strain BL21 Star(DE3)pLysS; the corresponding ~6 kDa peptides could be purified after induction with IPTG via Ni-chelate chromatography and were used to immunize rabbits (*Davids Biotechnologie; Regensburg, Germany*).

### Isolation of RNA, reverse transcription PCR, and northern blot experiments

500 ml of exponentially growing cells (~1 × 10^7^ cells/ml and 4–5 × 10^7^ cells/ml; direct cell counting using a Thoma counting chamber) or stationary cells (~2 × 10^8^ cells/ml) were collected by centrifugation and resuspended in 1 ml of Trizol™ each. After incubation for 10 min at room temperature 0.2 ml chloroform was added and the lysate was cautiously mixed. After centrifugation (12,000 × g, 15 min, 4°C) the water phase was collected, 0.5 ml ice-cold 2-propanol was added and precipitation was for 30 min or overnight at −20°C. RNA was collected by centrifugation as above and the pellet was washed with 1 ml of ice-cold 70% ethanol. The pellet was air dried, resuspended in 100 μl H_2_O and 90 μl *DNase I Incubation Buffer* plus 10 μl *DNase I* (both from *High Pure RNA Isolation Kit, Roche Diagnostics GmbH; Mannheim, Germany)* were added. After 15 min incubation at room temperature further processing, including a phenol/chloroform extraction and RNA precipitation, was as recommended in the *High Pure RNA Isolation Kit*.

To detect specific mRNA transcripts, mRNA was transcribed into cDNA using the *Super Script II reverse Transcriptase* protocol as suggested by the supplier (*Invitrogen GmbH; Karlsruhe, Germany*). The various cDNAs were amplified via PCR using different combination of primers which were designed for each gene of the flagellar operon; primers are given in Supplementary Figure S1. In each case a negative control without addition of cDNA was included; the positive control included PCR reactions using genomic DNA.

For Northern Blotting, RNA probes were labeled with digoxygenin using the *DIG Northern Starter Kit (Roche Diagnostics GmbH; Mannheim, Germany)*. Gel electrophoresis, northern blot, hybridization and detection was as recommended in the manufacturer's instructions.

### TEM analyses

Preparation of specimens by negative staining and for immuno-labeling was as described earlier (Näther et al., 2006; Rachel et al., 2010).

## Results

### Identification of *flaB0*, a third flagellar gene, in the genome of *P. furiosus*

**Figure 1:**
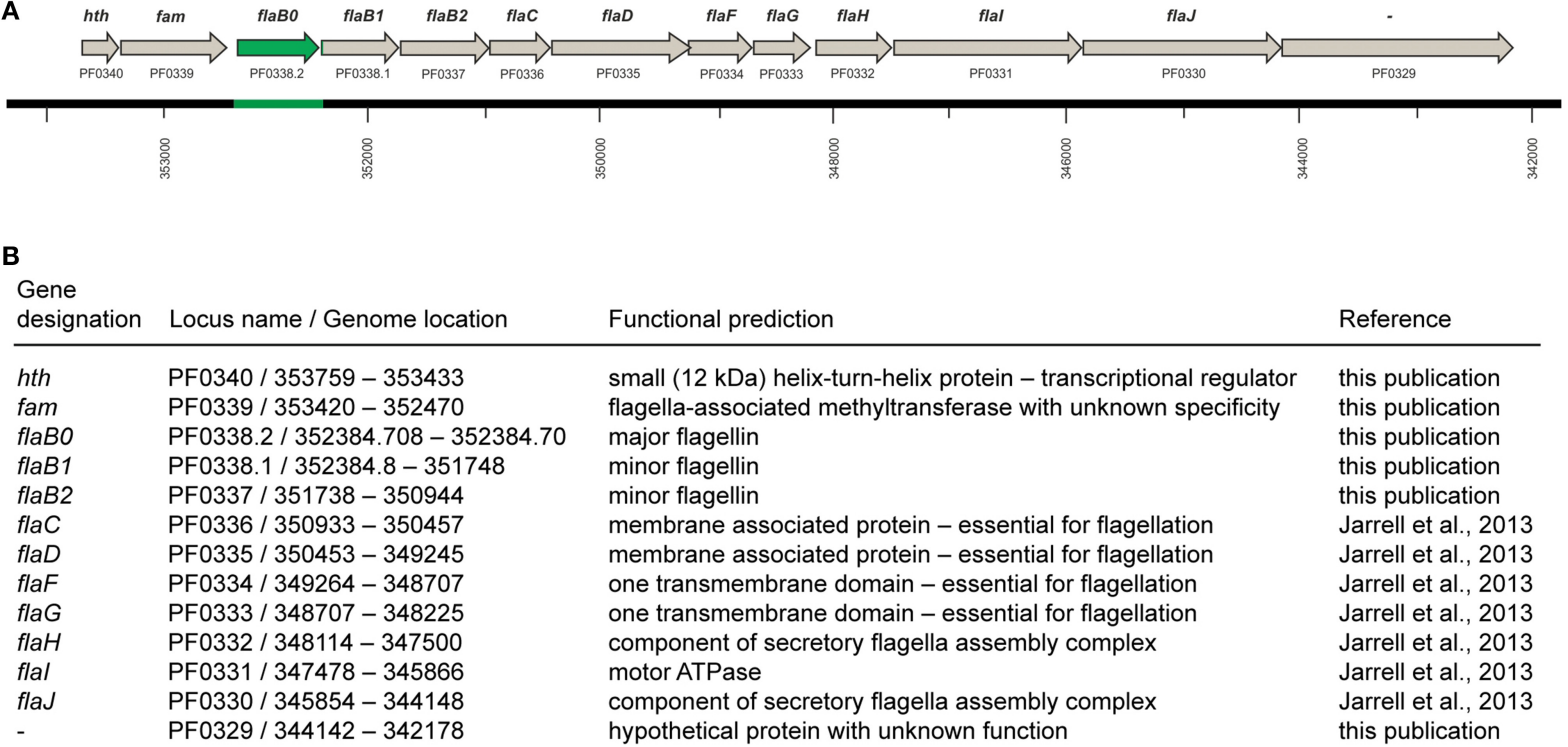
**The flagellar operon of *Pyrococcus furiosus***. **(A)** The revised flagellar operon with neighboring genes. The missing genomic sequence and the *flaB0* gene are marked in green. All genes are transcribed from the negatively oriented DNA strand, but are shown here from left to right for easier orientation. **(B)** Functional predictions of the genes shown in **(A)**.

Purification of flagella via isopycnic CsCl-gradient centrifugation and analysis by SDS-PAGE identified one major glycoprotein, whose N-terminal amino acid sequence was determined to be AVGIGTLIVF (Näther et al., 2006). Sequence alignments illustrated that this N-terminal sequence did not perfectly match to the annotated flagellins of *P. furiosus* or to any of the other proteins translated from the published genome (Robb et al., 2001). More precisely, the N-terminus of protein FlaB2 should read AIGIGTLIVF, but Edman degradation of the major flagellin never indicates any heterogeneity at position 2. In case of protein FlaB1 we found that the published sequence lacks the motif AIGIGTLIVFIAM, which is very highly conserved in all flagellins annotated in the publically available genomes of the genus *Pyrococcus*. However, this motif is encoded directly in front of the annotated *flaB1* gene but misses an upstream in-frame start codon. Based on these findings we decided to resequence the genome region that codes for the flagellins. Indeed, we identified a major mistake in that part of the published *P. furiosus* genome sequence: a total of 771 bp are missing. By combining this new sequence with the published genome (Robb et al., 2001), the sequence of the *flaB1* gene now contains a proper start codon and its N-terminus becomes highly similar to other flagellins. In addition, we detected another ORF coding for a third flagellin which we call *flaB0* to keep in line with the existing nomenclature. As a consequence, the flagellar operon of *P. furiosus* was revised (Figure 1). The missing genomic sequence containing the annotation of the *flaB0* gene/FlaB0 protein was submitted to NCBI BankIt; the corresponding GenBank number is KM892551.

### Flab0, the major flagellin of *P. furiosus*

All flagella isolated from *P. furiosus* by different methods (shearing, DDM treatment, Triton X-114 treatment according to Kalmokoff et al., 1988) over a period of nearly 10 years were composed of one major flagellin as indicated by SDS-PAGE. The finding that the N-terminal amino acid sequence of this protein unambiguously was AVGIGTLIVF suggests that the newly detected FlaB0 is the major flagellin of *P. furiosus* whereas FlaB1 and FlaB2 are only minor flagellins.

**Figure 2:**
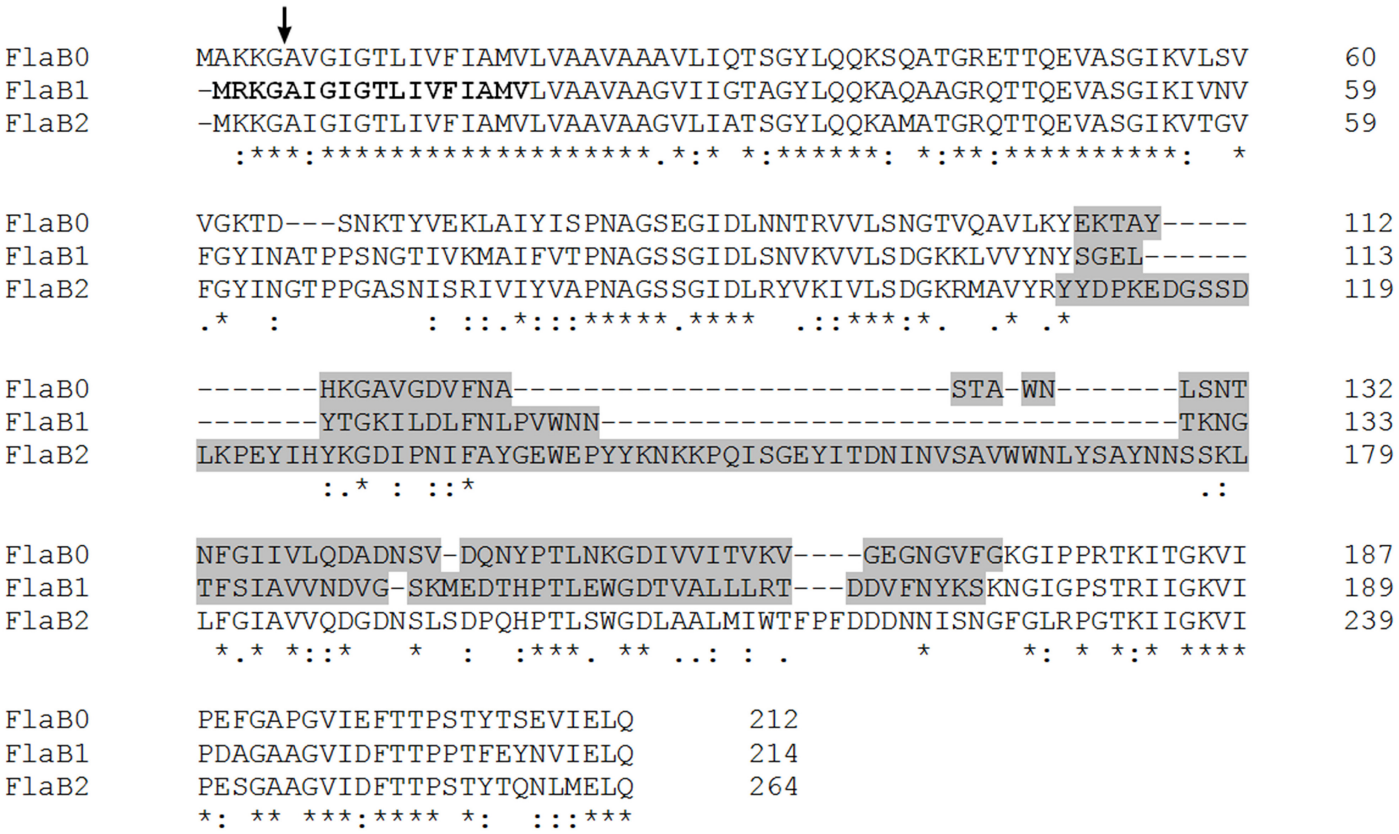
**Sequence alignment of the three *P. furiosus* flagellins**. Amino acid identities for the three proteins are indicated by asterisks (^*^); conservative amino acid exchanges are indicated by colons (:), and semi-conservative amino acid exchanges are indicated by dots (.). The arrow shows the signal peptidase processing site. Bold ladders represent the FlaB1 sequence correction resulting from the resequencing performed in this study. Regions indicated by gray sequences identify the least conserved central part of the proteins used to raise flagellin-specific antibodies (primers used for cloning are given in Materials and Methods).

To ask for the presence of the two minor flagellins in our flagella preparations, we raised specific antibodies against all three flagellins. Because of the highly conserved N- and C-terminal part of FlaB0, FlaB1, and FlaB2, we subcloned the unique central part of each flagellin (gray sequences in Figure 2) and used the peptides for immunization of rabbits. The resulting antisera had a low titer, especially for the FlaB2-peptide. Western blots (data not shown) using these antisera proved that all three flagellins are present in the protein band at around 30 kDa. In addition, purified antibodies were used to immuno-label flagella preparations and cells adherent to carbon-coated gold grids for TEM. Again, we could show that antibodies against sheared *P. furiosus* flagella detach adherent cells from their solid support as described earlier (Näther et al., 2006). Some single cells, however, remained on the grid and their flagella were clearly labeled over their whole length. In contrast, no signals were detected using any of the antibodies against the recombinant flagellin middle parts. Specific antibodies against FlaB1 and FlaB2 reacted mostly with the ends of purified flagella (data not shown).

### Denaturated flagella can repolymerize into filamentous structures

We furthermore asked if “native flagella” could be repolymerized from denatured flagellins, spontaneously without energy and without a template. For depolymerization a flagella preparation was denatured by addition of SDS and incubation at 100°C, followed by extensive dialysis. We found that heat treatment is necessary for complete denaturation, otherwise SDS-PAGE shows the presence of minor protein bands with molecular weights of ca. 60, 90, and >100 kDa. Two dimensional SDS-PAGE clearly proved that these bands could be dissociated into the ~30 kDa flagellin monomers (data not shown).

**Figure 3:**
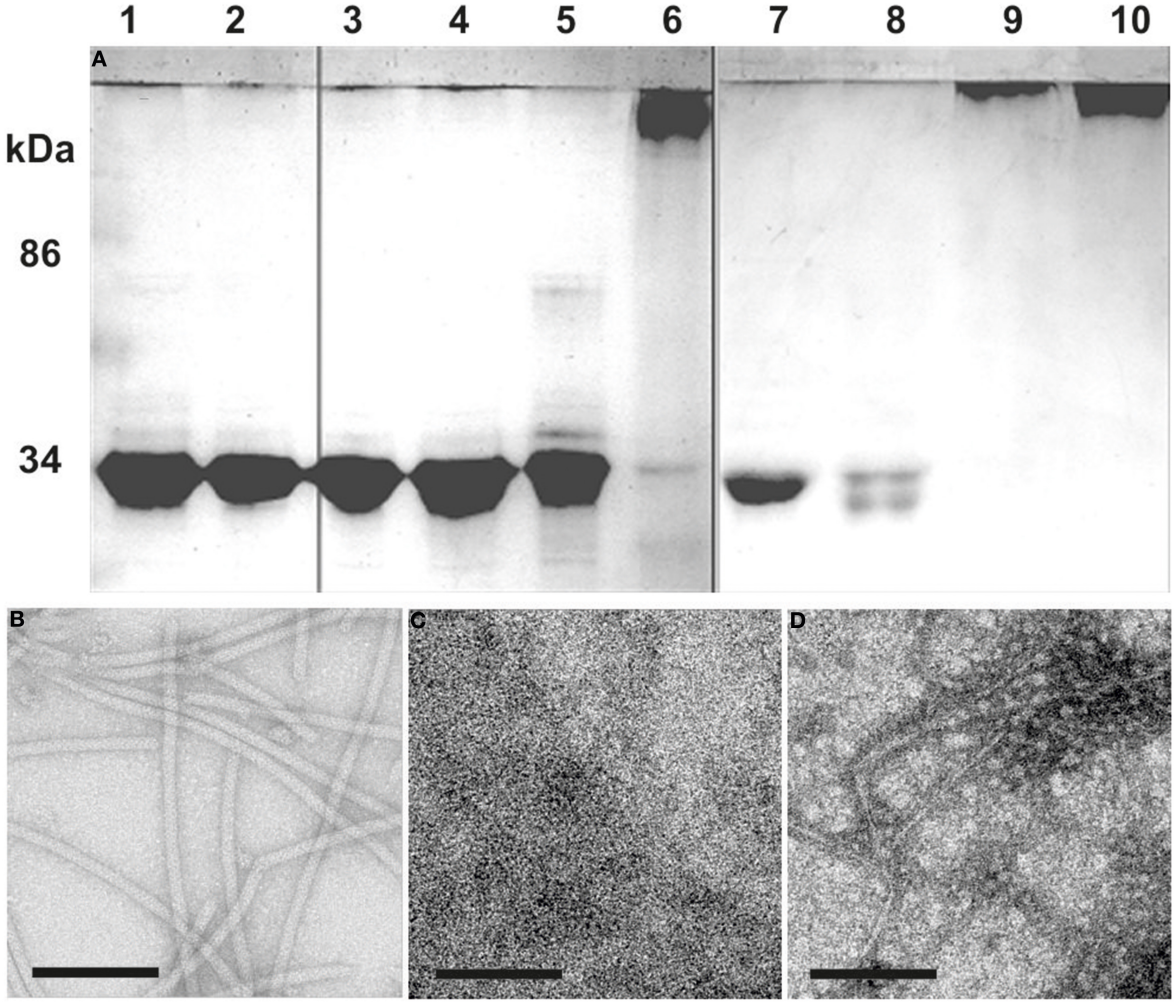
**Repolymerization of denatured flagella**. **(A)** SDS-PAGE: Flagella purified by CsCl-gradient centrifugation were denatured into monomeric flagellins by SDS and heat denaturation (lane 1). After extensive dialysis against 5 mM HEPES buffer only single flagellins were observed (lane 2). The denatured flagellins were used for polymerization assays at: 8°C (lanes 3 and 7), 37°C (lanes 4 and 8), 60°C (lanes 5 and 9), and 90°C (lanes 6 and 10). Analysis was done after 1 (lane 3–6) or 6 days (lane 7–10) of incubation. **(B–D)** show TEM analyses of: **(B)** the flagella preparation; **(C)** denatured flagellins (lane 2); **(D)** the result from a 90°C repolymerization after 1 day (lane 6). Size bars are 100 nm, each.

Flagellins from denaturated flagella were incubated at different temperatures to analyze their potential to repolymerize. Incubation for 1 day or longer at temperatures higher than 60°C resulted in aggregation of the ~30 kDa flagellins into high-molecular weight polymers, forming in part also filamentous structures as proven by TEM (Figure 3). Comparing these filaments to native flagella, we found the diameter to be smaller, and no helical ultrastructure was present. Addition of ATP to the samples had no influence on the formation of aggregates or filaments (data not shown).

### Conservation of *flaB0* in various *P. furiosus* strains

**Figure 4:**
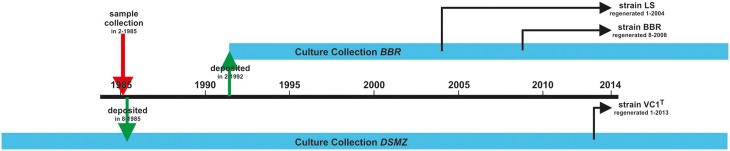
**History of the *P. furiosus* strains used in this study**. The type strain Vc1^T^ was deposited within 6 months after its isolation at DSMZ. Strains *P. furiosus* LS and BBR were regenerated at different times from our in-house culture collection and thereafter repeatedly grown and stored at 4°C.

The genome of *P. furiosus* has been reported to be dynamic (Bridger et al., 2012) — a feature of this hyperthermophile we experienced also in our Regensburg labs. Over the years, we have identified at least 2 strains differing from the original *P. furiosus* isolate whose origin/history is shown in Figure 4. The original strain named Vc1 was deposited as type strain DSM3638^T^ at the German Culture Collection (Deutsche Sammlung für Mikroorganismen und Zellkulturen, DSMZ) ca. 6 months after its isolation. The same isolate was repeatedly regrown (for ca. 7 years) from stocks stored at 4°C and deposited in 1992 in our Regensburg Culture Collection, (**B**akterien**b**ank **R**egensburg, BBR). Therefrom strain LS was regenerated in 2004 and was repeatedly regrown from stocks stored at 4°C. Another derivate, strain BBR was regenerated from our in-house culture collection in 2008 and repeatedly regrown from stocks stored at 4°C. The three strains of *P. furiosus*, namely Vc1^T^, LS, and BBR, differ with respect to their binding behavior to various surfaces if tested as described (Näther et al., 2006), they express different amounts of flagella and their cell morphology differs drastically (Bellack, 2011; data will be described in detail elsewhere).

**Figure 5:**
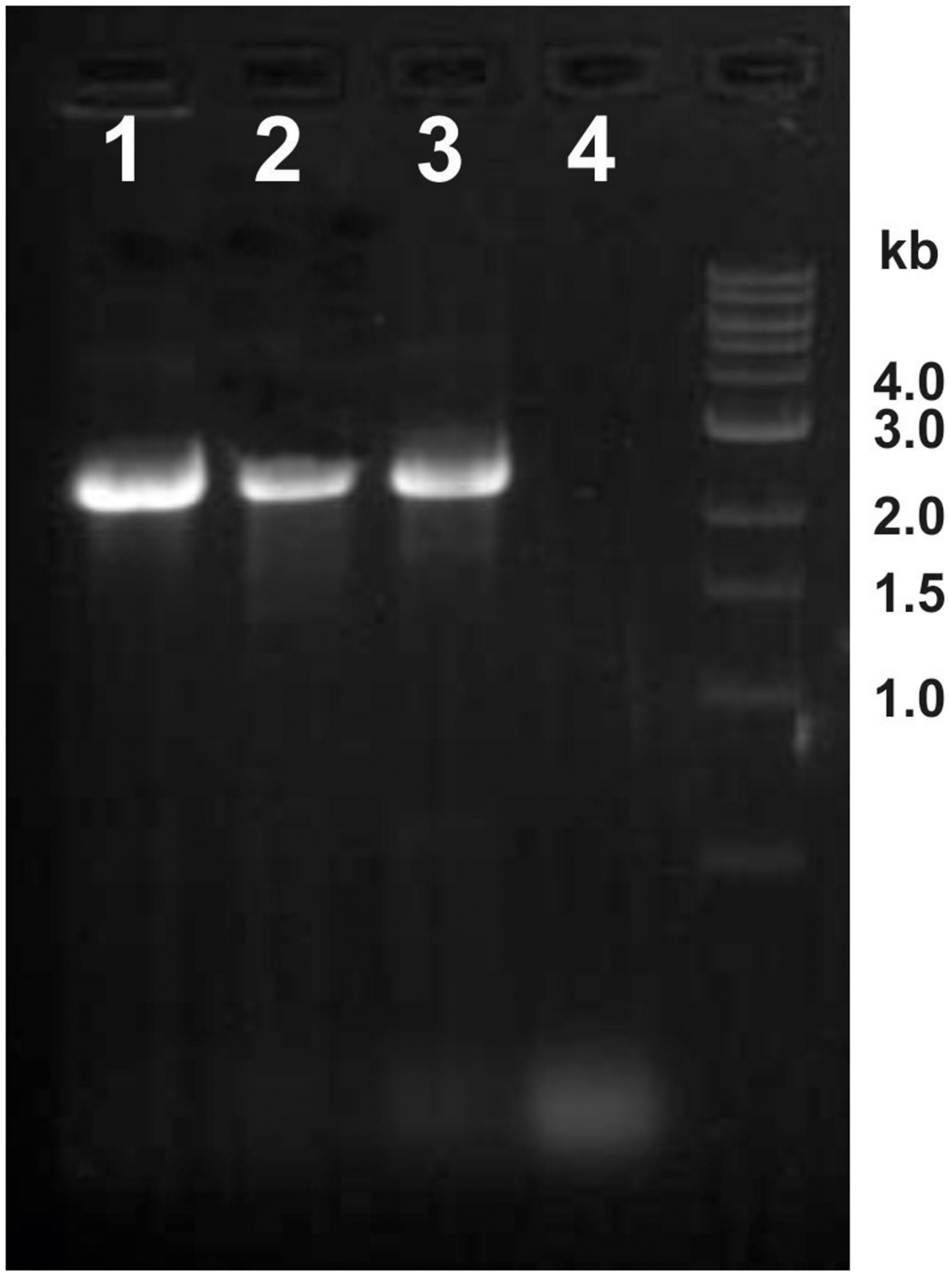
**Detection of the *flaB0* gene in three different *P. furiosus* strains**. Genomic DNA was isolated from the three strains *P. furiosus* Vc1^T^, BBR, and LS and used for PCR amplification with primers 353483_f_ and 351761_r_. Very clearly a ca. 2.5 kb amplificate was identified in all three strains; if *flaB0* would be missing (as in the original sequence) a ca. 1.7 kb amplificate would be expected. Lane 1, strain LS; lane 2, strain BBR; lane 3, strain Vc1^T^; lane 4, negative control.

We therefore asked if the newly discovered flagellin gene *flaB0* is conserved not only in the type strain but also in the two lab derivates. Hence, genomic DNA was isolated and primers 353483_f_ and 351761_r_ were used to amplify the region around *flaB0*. For all three strains a 2.5 kb fragment was amplified as expected for the presence of *flaB0* (Figure 5). As the primer numbers refer to the binding position in the public genome of *P. furiosus* (Robb et al., 2001), the fragment should be only 1.7 kb in length when no *flaB0* would be present. Genomic sequencing of the *flaB0* region confirmed the sequence we determined earlier for the missing 771 bp segment in all three strains (data not shown).

### Transcriptional analyses of the *P. furiosus* flagellar operon

We asked if all flagella-related genes of *P. furiosus* would be transcribed together, or if various smaller transcripts might exist. Transcripts were analyzed by PCR after reverse transcription of mRNA into cDNA, the positive control used genomic DNA instead. A negative control without addition of cDNA proved that in all cases only transcripts from mRNA were analyzed (data not shown).

**Figure 6:**
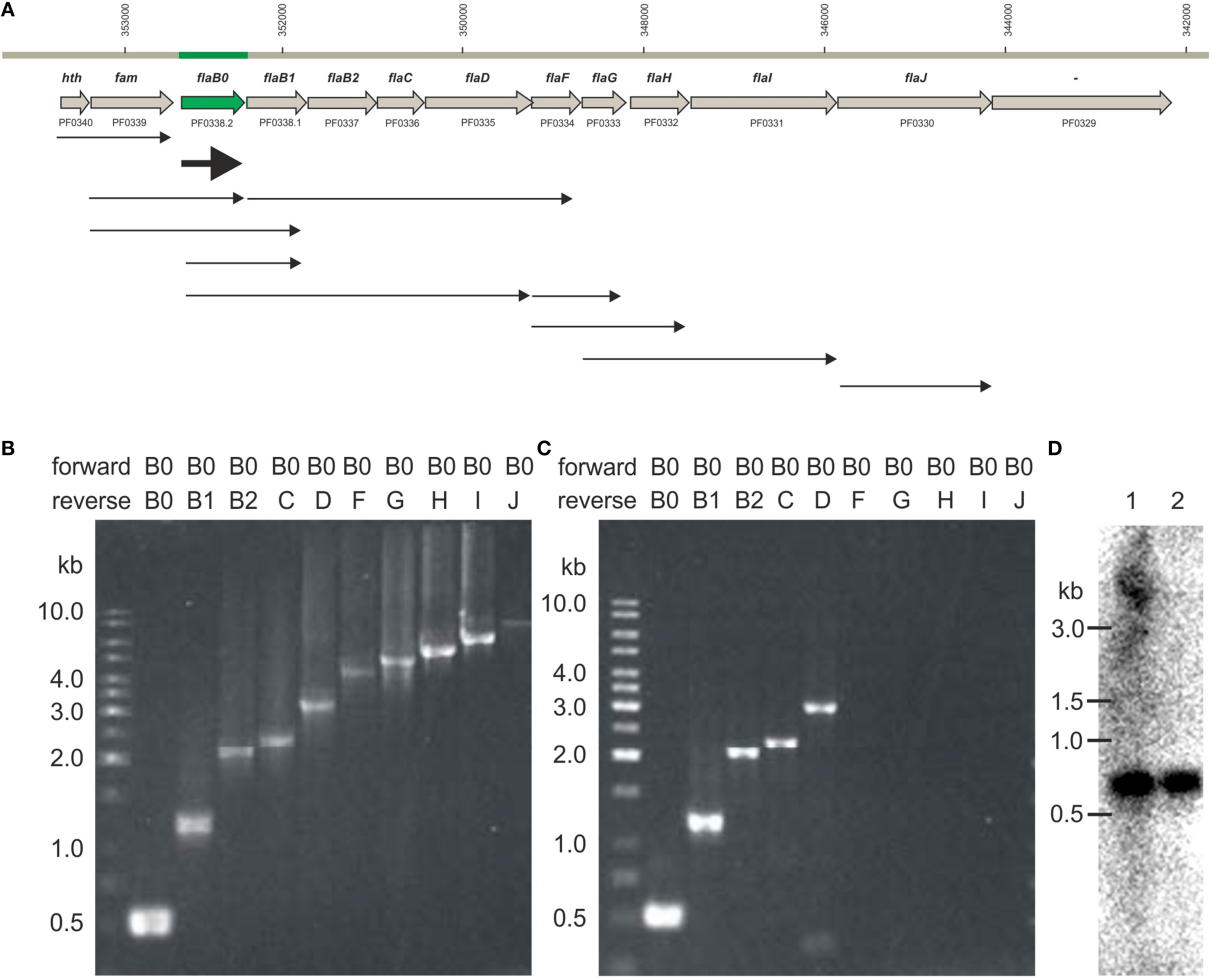
**Transcripts observed for the *P. furiosus* flagellar operon**. **(A)** Flagellar operon of *Pyrococcus furiosus* with neighboring genes in the upper part. All genes are transcribed from the negatively oriented DNA strand, but are shown here from left to right for easier orientation. Arrows in the lower part indicate cotranscripts identified via RT-PCR. **(B)** PCR data using genomic DNA as positive control; forward primer was Pfu-flaB0_f, reverse primers were Pfu-flaB0_r to Pfu-flaJ_r. **(C)** PCR data using cDNA after reverse transcription of isolated RNA and the same primers as given in **(B)**; (data for all other transcripts are found in Supplementary Figure S1). **(D)** Northern blot experiments using a *flaB0* probe. RNA was isolated from late exponentially growing cells (lane 1) and cells in stationary phase (lane 2) and separated in a denaturing agarose gel. The gel migration behavior of an RNA standard is indicated to the left.

**Figure 7:**
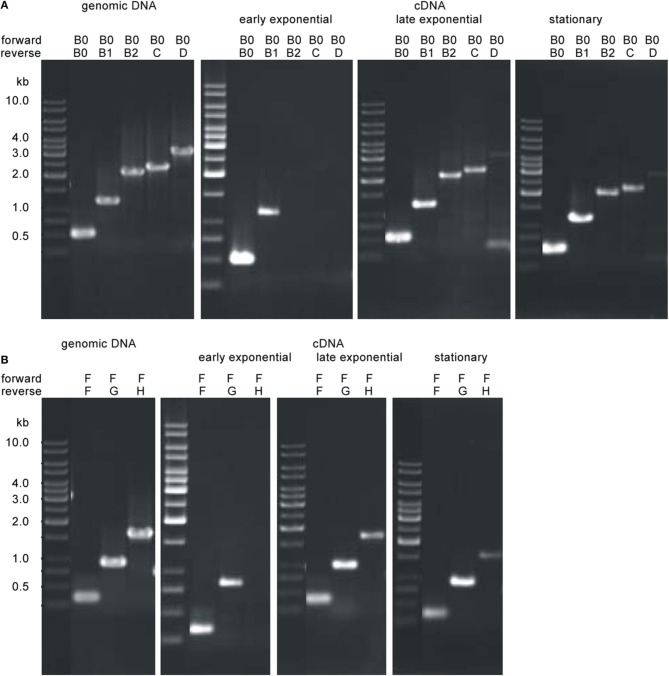
**Transcriptional analyses of the *P. furiosus* flagellar operon depending on the growth phase**. Total RNA was isolated from cells of early exponential growth phase (~1 × 10^7^ cells/ml; panel 2), late exponential growth phase (4–5 × 10^7^ cells/ml; panel 3) and stationary growth phase (~2 × 10^8^ cells/ml; panel 4) and transcribed into cDNA; transcripts were amplified by PCR using different sets of primers. PCR data using genomic DNA as positive control is shown in panel 1. Results are shown exemplarily for two different potential cotranscripts. **(A)** Analysis of the potential cotranscript *flaB0-flaD*. Forward primer was Pfu-flaB0_f, reverse primers were Pfu-flaB0_r to Pfu-flaD_r. **(B)** Analysis of the potential cotranscript *flaF-flaH*. Forward primer was Pfu-flaF_f, reverse primers were Pfu-flaF_r to Pfu-flaH_r.

We detected different length cotranscripts for each of the genes of the flagellar operon with exception of *flaJ* were only the single gene transcript was found (Figure 6A). The original data using the different primers are shown exemplarily for *flaB0* in Figures 6B,C, all other data are given in Supplementary Figure S1. Several transcripts including *flaB0* were found whereof the largest with ca. 3.1 kb contained all three flagellins, *flaC*, and *flaD*. Besides, various transcripts for the genes *flaF-flaI* were detected. Interestingly, we found a transcript containing *hth* and *fam* whereas *flaJ* and *PF0329* were never part of a cotranscript. Analyses of RNA of cells from different growth phases showed that the transcripts changed over time; the original data are shown exemplarily in Figure 7. In early exponential phase, only few short transcripts were present compared to late exponential and stationary phase indicating that the flagellar operon is transcribed only to a limited degree in early exponential growth phase.

Northern blot experiments using RNA isolated from late exponentially growing cells showed the existence of a prominent ca. 600 bp long transcript using a probe for *flaB0*. In addition a much less prominent smear above ca. 4 kb was detected (Figure 6D). For cells in the stationary growth phase, only the ~600 bp long *flaB0* transcript was detected.

## Discussion

### Difficulties to clone *flaB0* might have prevented its identification in genome sequencing

The genome of *P. furiosus* was one of the first archaeal genomes to be sequenced (Robb et al., 2001). In those “old days” of genome sequencing the shotgun cloning and sequencing approach — first used by the Venter lab to determine the *Haemophilus influenzae* genome (Fleischmann et al., 1995) — was the only way to obtain reliable data. A general problem with this approach is the fact that some genes are difficult to clone or might be even toxic for the host, normally *Escherichia coli*. In our studies, we found that *flaB0* could not be cloned into *E. coli* using standard approaches. Cloning in a vector system with expression under the strong T7 polymerase promoter as used e.g., for cloning of flagellins of *Methanococcus voltae* (Bayley and Jarrell, 1999) or in the IMPACT system (*intein-mediated purification with an affinity binding tag*) failed since the protein turned out to be toxic. We furthermore experienced problems with subcloning parts of *P. furiosus* flagellin genes, which was especially true for *flaB0*. Only the middle part of *flaB0* could be cloned, but not the N- and C-terminal regions. Hence, we suggest that these problems in cloning might also have happened during the original genome sequencing.

The only way we could obtain the *flaB0* sequence was to sequence directly from genomic DNA via primer walking. Since reading lengths of >600 bp were very difficult to obtain in the early days of genomic sequencing it is not too surprising in retrospect that *flaB0* was not detected in the original published *P. furiosus* genome sequence (Robb et al., 2001).

### FlaB0 is the major flagellin of *P. furiosus* flagella

The here newly described gene *flaB0* codes for the major flagellin of *P. furiosus*, whereas FlaB1 and FlaB2 are only minor flagellins. This statement is supported by the fact that one major glycoprotein of ca. 30 kDa was identified in all flagellar preparations isolated over a period of nearly 10 years; the N-terminal sequence of this protein read as AVGIGTLIVF which can be matched clearly to FlaB0. There was no heterogeneity at position 2 and the presence of FlaB1 and FlaB2 was proven only by use of specific antibodies. In further experiments we were able to show that antisera raised against the least conserved part of the flagellins FlaB1 and FlaB2 labeled CsCl-gradient purified flagella mostly on their ends, whilst an antiserum raised against purified flagella reacted much stronger over the whole length of flagella.

### Repolymerization of denatured flagella

Flagellins derived from SDS- plus heat-denatured flagella could clearly be repolymerized into smaller aggregates and fibrillar structures via simple heat treatment. The ultrastructure and diameter of such fibrils differs obviously from that of purified flagella. This, however, is not too surprising if one takes into account that for flagella assembly most likely a platform containing (at least) the proteins FlaC, FlaD, FlaF, and FlaG is necessary *in vivo* (see Jarrell et al., 2013 and references therein). In addition this process is supposed to require ATP; in our hands repeated ATP addition to the *in vitro* repolymerization assays, however had no effect.

### The flagellar operon of various *P. furiosus* strains

Transcription of genes *pf0329* to *pf0340* is from the negatively oriented DNA strand, whilst the neighboring genes are transcribed form the positively oriented strand. Operon prediction using the Prokaryotic Operon DataBase (ProOpDB; Taboada et al., 2012) reveals two operons in this region encoding PF0340-PF0339 and PF0338-PF0329, respectively. Therefore, we analyzed this part of the genome for transcription including the flagellar operon neighboring genes *pf0329* and *pf0340*. Both, our RT-PCR experiments and Northern Blot analyses show that there is not a single cotranscript detectable for the genes inside the flagellar operon of *P. furiosus*, starting at *flaB0* (or even *pf0340*) and ending with *flaJ* (or *pf0329*). Rather, various cotranscripts were detected. Single transcripts were observed for *flaB0* and *flaJ*. From these results we conclude that the flagellar operon is composed of genes *pf0340-pf0330*, whereas the gene encoding the hypothetical protein PF0329 is not a part of the operon.

Possible explanations for the existence of various cotranscripts we identified are as follows. *fam-flaB0, fam-flaB1*: the specificity of the postulated methyltransferase is unknown; it potentially could modify FlaB0 and FlaB1 (and also FlaB2). Analyzing the publically available genomes of the genus *Pyrococcus* for the presence of an ortholog of the *P. furiosus* methyltransferase, we found the respective gene in all species directly upstream of the flagellin genes, supporting our hypothesis that the enzyme acts on flagellins. *flaB0-flaD, flaB1-flaF*: FlaC, FlaD (and FlaE, which is not present in *P. furiosus*) are argued to be necessary for flagella assembly (see e.g., Schlesner et al., 2009); therefore, a coexpression with the flagellins would be expected. *flaF-flaG*; *flaF-flaH*; *flaG-flaI*: FlaF and FlaG have been argued to be essential for expression of flagella (Jarrell et al., 2013). Since no direct data for the function of those proteins are available, any argument about cotranscription or direct interaction of encoded proteins would be pure speculation. FlaH, FlaI, and FlaJ are probably part of the secretion and motor complex of archaeal flagella (Jarrell et al., 2013). Structural and genetic studies of the ATPase FlaI of *S. acidocaldarius* revealed that the protein forms a hexameric crown-like ring; its conformational changes and interactions with membrane lipids and binding partners (mostly FlaJ) regulate assembly and rotation of flagella (Reindl et al., 2013). Hence we expected cotranscription of the corresponding genes. However, our results showed that *flaJ* is transcribed only as a single gene regardless of the growth phase. In contrast, the cotranscripts described herein differed depending on the growth phase; these findings are in line with our electron microscopic studies of *P. furiosus* cells showing that flagella are assembled particularly in late logarithmic phase (early exponential cells possess no or only few flagella; data not shown).

The absence of one single flagellar transcript is supported by data for other Archaea: a very good overview was given by Thomas et al. (2001) (see especially Figure 4, therein). In all cases, one or more major transcripts encoding the flagellins — FlaA and/or FlaB proteins — have been identified by Northern blots. Minor transcripts, argued to code for additional proteins FlaC to FlaJ have been found in all of these cases; transcripts not starting with *flaA* or *flaB*, however have not been analyzed. Also for *Sulfolobus solfataricus* a major transcript, encoding the flagellin FlaB has been found (Szabo et al., 2007), but again transcripts encoding further genes in the flagellar operon have not been characterized.

Because the three *P. furiosus* strains presented in this study show differences in the number of flagella and adhesion properties the question arose if their flagellar operons might be different and, most notably, if the *flaB0* gene is conserved. The genome of *P. furiosus* is known to be highly dynamic as proven for the genetically tractable strain COM1 (Bridger et al., 2012) and different *Pyrococcus* strains originated from environmental samples (e.g., Escobar-Paramo et al., 2005; White et al., 2008). COM1 is derived from strain DSM3638^T^ by targeted gene disruption of the *pyrF* locus (Lipscomb et al., 2011), it possesses 45 full or partial insertion sequences compared to 35 in strain DSM3638^T^, resulting in inactivation of 13 genes. In addition alterations in 102 of 2134 predicted genes were observed, together with major chromosomal rearrangements, deletions etc. (Bridger et al., 2012). Despite these proven changes in the genome, we found that *flaB0* is well-conserved in all three *P. furiosus* strains described in this study supporting our data that *flaB0* encodes the major *P. furiosus* flagellin. Differences in flagellation and adherence, therefore, might be caused by alterations in other regions of the genome and/or regulatory effects. In this context, we note to have shown earlier that flagella contribute to adhesion (Näther et al., 2006), but we also are aware of the fact that other archaeal cell surface appendages like pili (Jarrell et al., 2011), fibers (Müller et al., 2009), fimbriae (Thoma et al., 2008), and hami (Moissl et al., 2005) at least can contribute to adhesion to various surfaces.

## Author contributions

Daniela J. Näther-Schindler, Simone Schopf, Annett Bellack, Reinhard Rachel, and Reinhard Wirth designed the study and analyzed the data. Research was performed by Daniela J. Näther-Schindler, Simone Schopf, and Annett Bellack. Annett Bellack and Reinhard Wirth wrote the paper; all authors agreed to the final version.

### Conflict of interest statement

The authors declare that the research was conducted in the absence of any commercial or financial relationships that could be construed as a potential conflict of interest.
