# Supplementary material for: Pyrococcus furiosus flagella: biochemical and transcriptional analyses identify the newly detected flaB0 gene to encode the major flagellin
Source: Front Microbiol. 2014 Dec 11;5:695. doi: 10.3389/fmicb.2014.00695 (PMC4263178; doi:10.3389/fmicb.2014.00695)

**Supplementary Material**

**Supplementary Figure S1:**

**Transcription analyses of all genes in the *P. furiosus* flagellar operon.** In each case the left panel shows PCR data using genomic DNA as positive control; the right panel shows PCR data using cDNA after reverse transcription of isolated RNA. Data for *flaB0* containing transcripts are shown in Figure 6 of the main manuscript.

The forward and reverse primers used are indicated; their sequences are as follows:

Pfu-flaB0_352384.680_f GAACCCTTATAGTGTTTATTGCCAT

Pfu-flaB0_352384.181_r AATACCCTTACCGAAAACTCCATT

Pfu-flaB1_351922_f CGACACTGTTGCACTGCTTC

Pfu-flaB1_351749_r CACTGCAGTTCTATCACATTATACTCA

Pfu-flaB2_351643_f TGGATATTTGCAGCAGAAGG

Pfu-flaB2_350944_r TCATTGAAGTTCCATTAAGTTTTGG

Pfu-flaC_350933_f ATGCCACTCGACTTCCTTTC

Pfu-flaC_350734_r ATGTTGTCAATGCTTATCTTTATCC

Pfu-flaD_350337_f GGATTCCTTAACCCCTAGACAA

Pfu-flaD_349952_r GCTCTTCAATCCTGGCTTTG

Pfu-flaF_349157_f GTATGCAGCATGGGACAGTG

Pfu-flaF_348859_r TTTGGAAGTAGTCCCCAGGT

Pfu-flaG_348647_f AGTACGGTTGCTGGAGCATT

Pfu-flaG_348272_r GAACAACGACGATTCTGTGTG

Pfu-flaH_348030_f GCAAGCTATGTTTCAAGCCAAT

Pfu-flaH_347532_r CCACCTTGGGTTCGACTCTA

Pfu-flaI_347457_f CACTTGAAGATGCCATGAGAAG

Pfu-flaI_346676_r TGTCCCCCATGCAACTAACT

Pfu-flaJ_345822_f GGCAGACATAGACCCCAAAG

Pfu-flaJ_345124_r CCAACTTCAGCTGCCAAGAT


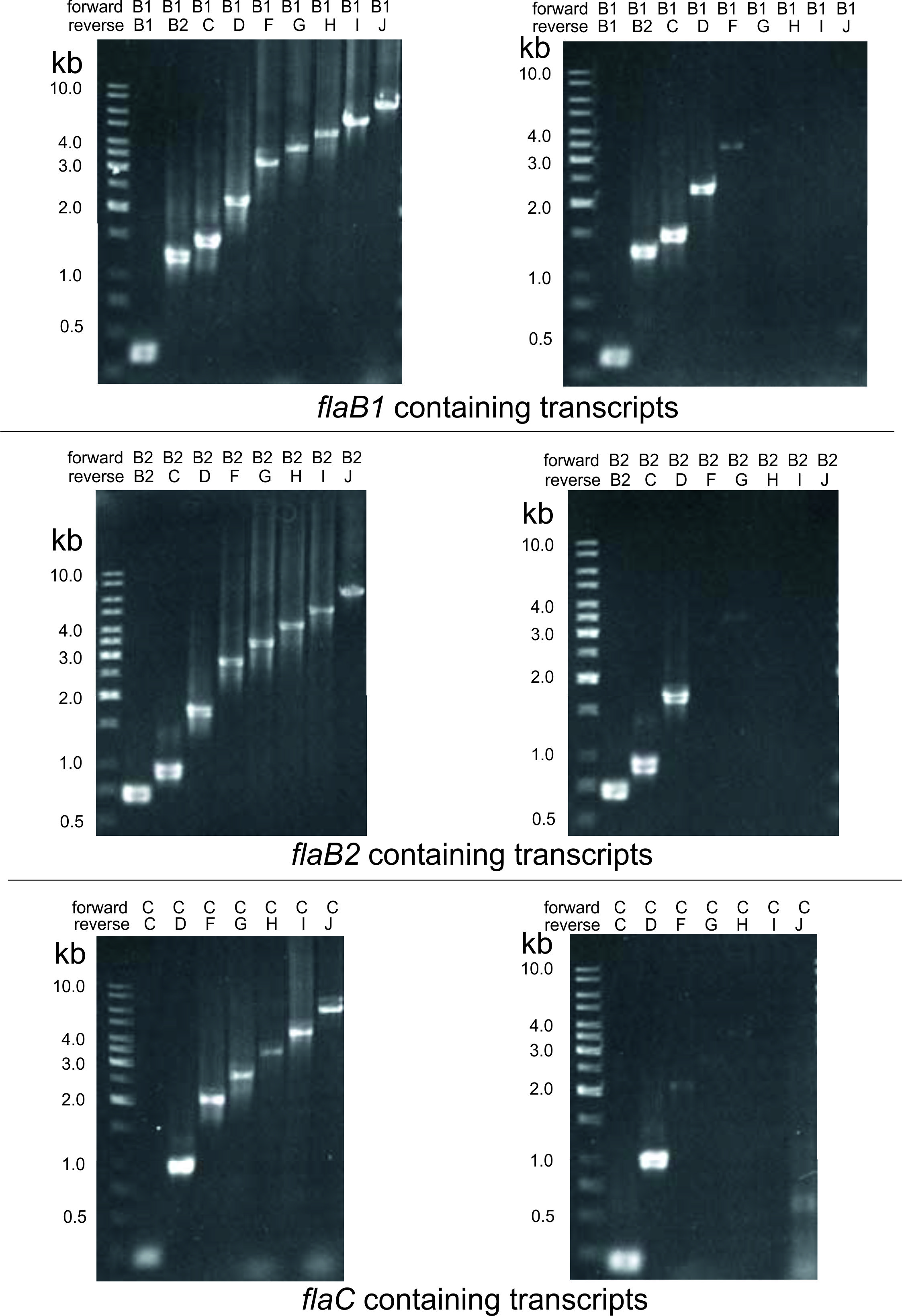


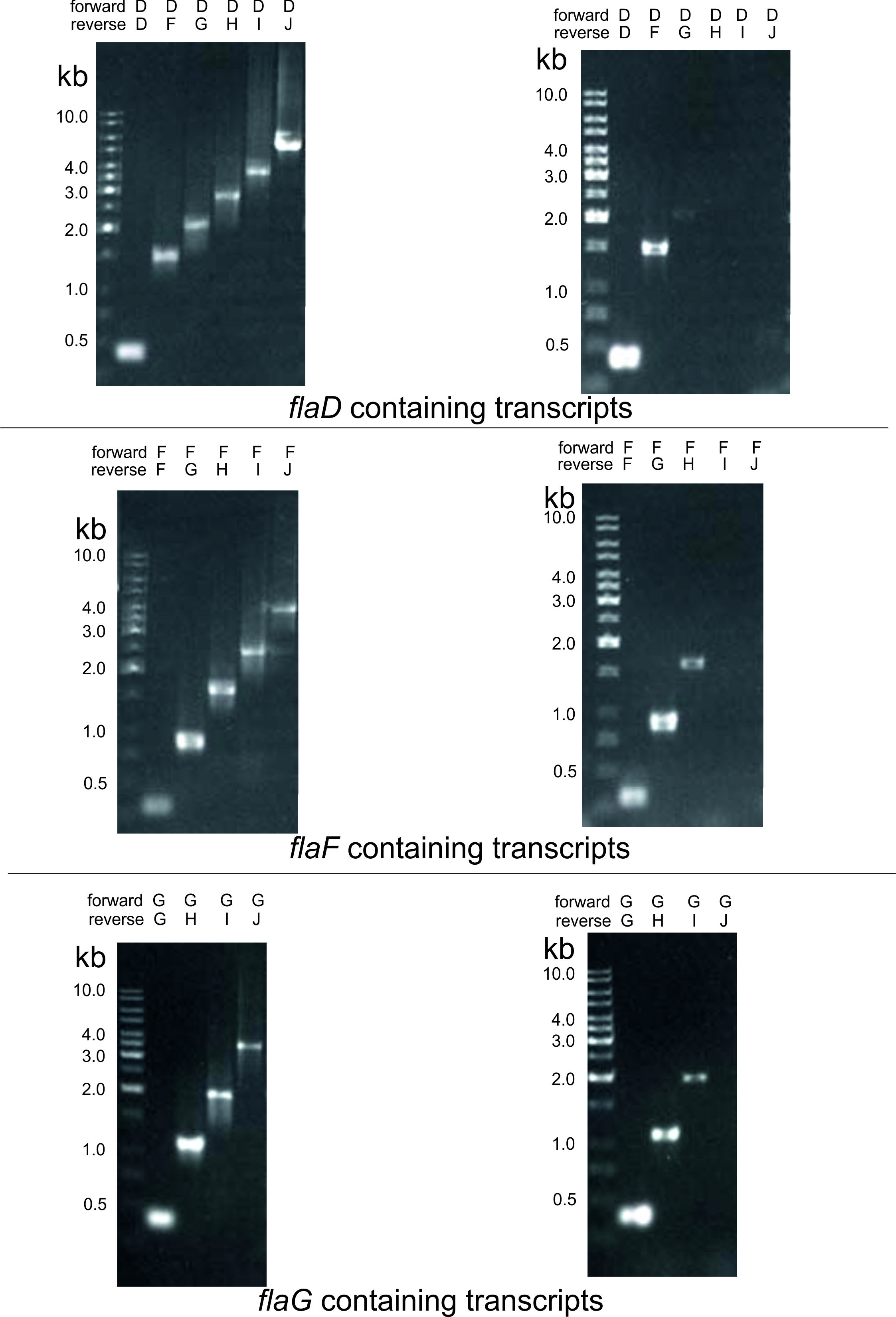


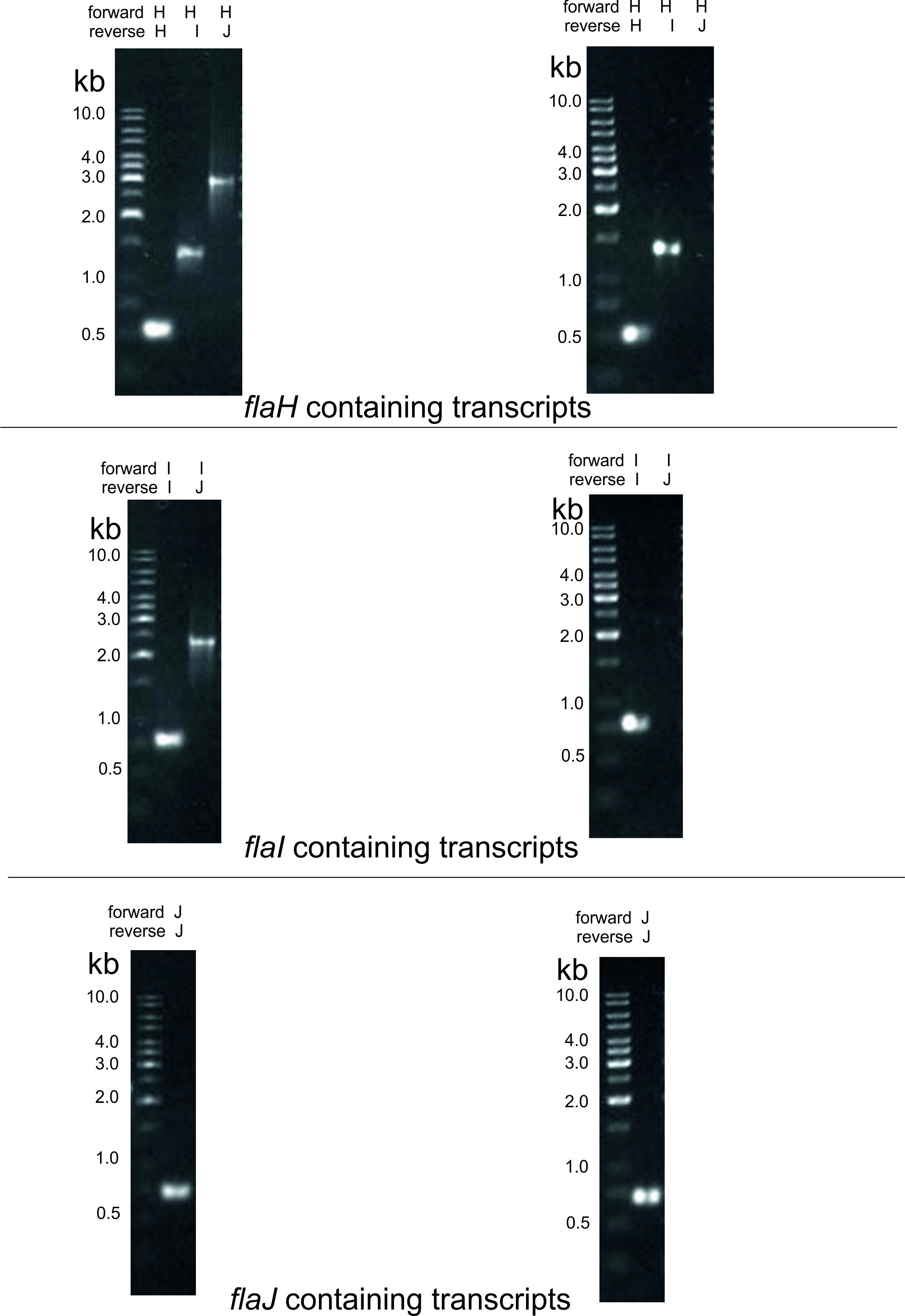

Supplement: Supplementary file 1 [file DataSheet1.DOCX]
